# Modelling the effects of immigration on the re-introduction of onchocerciasis

**DOI:** 10.1186/s13071-025-07213-z

**Published:** 2026-01-14

**Authors:** Jacob N. Stapley, Maria-Gloria Basáñez, Aditya Ramani, Martin Walker, Jonathan I. D. Hamley

**Affiliations:** 1 Department of Infectious Disease Epidemiology, MRC Centre for Global Infectious Disease Analysis and London Centre for Neglected Tropical Disease Research, School of Public Health, Imperial College London, 90 Wood Lane, London, W12 0BZ UK; 2 Department of Pathobiology and Population Sciences, Royal Veterinary College, Hawkshead Lane, Hatfield, Hertfordshire, AL9 7TA UK; 3 Multidisciplinary Center for Infectious Diseases, University of Bern, Bern, Switzerland; 4 Department of Visceral Surgery and Medicine, Inselspital, Bern University Hospital, University of Bern, Bern, Switzerland

**Keywords:** Onchocerciasis, Ivermectin, Immigration, Infection importation, Post-treatment surveillance, Post-elimination surveillance, Microfilarial prevalence, Anti-Ov16 seroprevalence, Stochastic modelling

## Abstract

**Background:**

Onchocerciasis is a filarial neglected tropical disease targeted by the World Health Organization for elimination (interruption) of transmission (EOT), principally by mass drug administration (MDA) with ivermectin. Variable effectiveness and success of MDA, among other factors, has led to a markedly heterogeneous contemporary spatial landscape of endemicity and transmission, with some foci having achieved or nearing EOT, while in others, transmission persists despite decades of MDA or has only recently been identified. Communities reaching EOT or free from infection are thus vulnerable to re-introduction of infection imported by immigrants from areas with ongoing transmission.

**Methods:**

We use the stochastic, individual-based EPIONCHO-IBM transmission model to quantify the risk of transmission persistence resulting from importation events and characterise the dynamics of ensuing onchocerciasis outbreaks in terms of microfilarial prevalence (in all ages) and anti-Ov16 seroprevalence (in children aged 5–9 years) in infection-free communities with local populations of black fly vectors.

**Results:**

We show how the vulnerability of infection-free communities depends on their population size, the local annual biting rate (ABR, number bites/person/year) and the magnitude of importation events, defined by the number of immigrants arriving in the community and their worm burden. We show that small communities with modest ABRs are particularly vulnerable to transmission persistence following importation, with risk exacerbated by the magnitude of infection importation. We illustrate that onchocerciasis outbreak dynamics can be protracted, with seroprevalence in children often taking substantially longer than the currently recommended 3–5 years of post-treatment surveillance (PTS) to exceed 5%.

**Conclusions:**

Our findings highlight the vulnerability of infection-free communities to introduction/re-introduction of infection and suggest that proposed PTS durations may need to be extended and complemented with additional surveillance activities and migration studies to detect and respond robustly to nascent outbreaks and sustain elimination.

**Graphical Abstract:**

**Figure:**
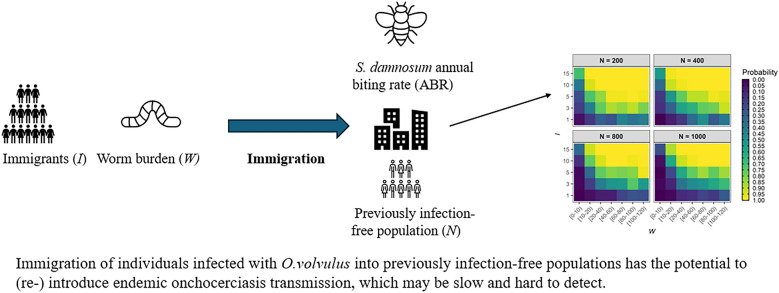

**Supplementary Information:**

The online version contains supplementary material available at 10.1186/s13071-025-07213-z.

## Background

Onchocerciasis (river blindness), a filarial disease caused by infection with the parasitic nematode *Onchocerca volvulus*, is transmitted among humans via bites of female *Simulium* black fly vectors which breed in fast-flowing rivers and rapids. Despite decades of interventions, onchocerciasis remains a substantial public health problem, particularly in sub-Saharan Africa (SSA), where more than 99% of the cases occur [1]. Currently, 28 countries in SSA remain endemic [2]. The Global Burden of Disease (GBD) Study estimated that, in 2021, approximately 20 million people (95% uncertainty interval [95% UI] = 18–22 million) were infected, and that the disease was responsible for 1.26 million (95% UI = 0.75–1.90 million) disability-adjusted life years (DALYs) [3]. Clinical manifestations leading to disease burden range from skin and ocular pathologies [4] to neurological involvement (onchocerciasis-associated epilepsy, OAE [5, 6]) and excess mortality (due to blindness [7], infection load [8, 9] and OAE [10]).

In its 2021–2030 road map for neglected tropical diseases (NTDs), the World Health Organization (WHO) proposed that elimination (interruption) of transmission (EOT) be verified in 12 (approximately one third of) endemic countries by 2030, mainly by implementing yearly mass drug administration (MDA) with ivermectin [11]. To date, four countries in Latin America (Guatemala, Mexico, Colombia and Ecuador) and one in West Africa (Niger) have been verified by WHO as having achieved EOT [12, 13]. In 2024, it was estimated that at least 248 million people in SSA required MDA, not including areas where transmission status is still unknown [12].

Vector control began in the mid-1970s under the Onchocerciasis Control Programme in West Africa (OCP, 1975–2002), covering 11 countries. Ivermectin MDA was used to complement vector control in core areas or as the sole intervention in extension areas beginning in the late 1980s [14]. The remaining endemic countries in SSA started MDA in the late 1990s under the African Programme for Onchocerciasis Control (APOC, 1995–2015) [15]. At the closure of APOC, national onchocerciasis control and elimination programmes became the responsibility of endemic countries, with the support of the Expanded Special Project for Elimination of Neglected Tropical Diseases (ESPEN).

Although many foci are deemed to be close to reaching EOT, infection persists in others despite decades of intervention and increased treatment frequency—particularly in those which were highly endemic before control started [16]. Elsewhere, interventions have made slow progress because of conflict or civil unrest, epidemic or pandemic outbreaks (e.g. Ebola, COVID-19), or co-endemicity with loiasis (which hampers routine ivermectin MDA and reduces adherence because of potential severe adverse events and/or fears of such events), or because interventions have only recently started or not yet begun in previously deprioritised low-endemicity areas or in those with previously unidentified transmission [12, 17–21]. Although onchocerciasis, as a river-associated vector-borne disease, is inherently focal, variable intervention implementation, duration and success have contributed to a contemporary markedly heterogeneous landscape, as indicated by geospatial and transmission dynamics modelling studies [22–24].

This heterogeneity presents a challenge to national programmes targeting EOT at a national level [24]. Not only can persistent foci prevent national progress towards verification, but movements of people and vectors can spread infection to foci where *O. volvulus* has been eliminated or would otherwise not be self-sustaining [25]. Black fly nuisance and onchocerciasis-associated morbidity (which are likely greater in former highly endemic areas with current persisting infection) [26] may not be the sole reasons for human migration, but they are contributory factors, particularly in the context of marginalised populations with limited economic opportunities.

Characterising migration patterns among rural SSA communities has been recognised as crucial to better understanding disease spread and intervention efficacy [27], particularly regarding malaria [28].

Several epidemiological studies have shown that in some communities nearly free of onchocerciasis, *O. volvulus* has been re-introduced by infected immigrants potentially harbouring high microfilarial loads, or that movement of uninfected people into onchocerciasis-endemic areas has led to onchocerciasis outbreaks. Such situations may arise as a result of, for instance, displacement due to conflict [29, 30], seasonal activities following agricultural work patterns (i.e. seasonal labourers) [31] or, as explored in this paper, a singular immigration event by which individuals from an endemic area settle permanently in a different, infection-free area [32, 33]. Given the protracted nature of the incubation and pre-patent periods of *O. volvulus*, newly established infections may take some time to become apparent [34].

Cross-border migration further complicates elimination efforts due to inconsistent inter-governmental cooperation and inter-country variation in intervention implementation [17, 35], especially in regions experiencing climate-driven natural disasters and/or conflict leading to population displacement [36].

Current WHO guidelines recommend stopping MDA when the seroprevalence of IgG4 antibodies to the *O. volvulus* Ov16 antigen is below 0.1% (at the upper 95% confidence limit) in children aged < 10 years, and the prevalence of infective flies is below 0.05% (also at the upper 95% confidence limit) [37]. This is followed by 3–5 years of post-treatment surveillance (PTS), which includes additional anti-Ov16 seromonitoring in children and xenomonitoring of black fly vector populations to detect infection resurgence or confirm EOT. If the latter is confirmed, a period of post-elimination surveillance (PES) follows (of indeterminate duration) [37]. Foci which have reached EOT remain vulnerable to the re-introduction of infection by infected immigrants during the PTS and PES periods and beyond, particularly if the ecological, environmental and socio-economic conditions that favoured transmission in the first place have not appreciably changed. Although early modelling work investigated the risk and dynamics of resurgence following cessation of vector control [38], little work has been undertaken on understanding the vulnerability of infection-free communities to imported infection and the ensuing dynamics of *O. volvulus* outbreaks. The slow, often undetectable early dynamics of recently introduced/re-introduced *O. volvulus* transmission motivate our (scenario) investigation of how ecological, epidemiological, demographic and immigration conditions would influence outbreak temporal trends and detectability and be critical to informing the design of robust surveillance strategies following EOT.

Here, we use the stochastic, individual-based model of onchocerciasis transmission, EPIONCHO-IBM [39], to (1) explore in silico the vulnerability of ‘archetypal’ communities free of onchocerciasis to *O. volvulus* introduction/re-introduction of infection as a result of single importation events, effected by the arrival of immigrants from areas with ongoing transmission, and (2) investigate the dynamics of subsequent outbreaks. We estimate the probability that immigration events—characterised by varying numbers of immigrants with varying infection intensity (adult worm burden)—will lead to persistent (endemic) infection and quantify outbreak growth rates in terms of microfilarial prevalence (in all ages) and anti-Ov16 seroprevalence (in children). We discuss the implications of our findings for verification of EOT, the suitability of the proposed duration of PTS, and the potential need for enhanced active surveillance and migration studies during the PTS and PES periods.

## Methods

### EPIONCHO-IBM

We used our stochastic, individual-based model EPIONCHO-IBM, parameterised for savannah onchocerciasis [39]. The model tracks the number of adult worms (*W*) and skin microfilariae in each individual host, their anti-Ov16 IgG4 antibody serostatus [40] and the mean number of infective (L3) larvae per (*Simulium damnosum* sensu lato [s.l.]) female fly. The human population is assumed to be stable with births balancing deaths, with both human and worm populations having balanced sex ratios. Individual exposure to black fly bites depends on age, sex [41], and an individual-specific exposure factor drawn from a gamma distribution with shape = rate parameter =  *k*_*E*_, ensuring that bites are distributed among hosts with mean exposure equal to the annual biting rate (ABR). Transmission is regulated by density-dependent processes in both humans and vectors, with the degree of exposure heterogeneity determined by *k*_*E*_. We used *k*_*E*_ = 0.3 throughout the analyses, as this value has previously been shown to provide the best fit for the ABRs investigated [39], but explored the impact of increasing exposure heterogeneity (*k*_*E*_ = 0.2) for an illustrative scenario. Each *k*_*E*_ value has an associated set of density-dependent parameters assumed to operate upon parasite establishment within humans [6, 39]. Adult worms and microfilariae have Weibull-distributed survival times, with mean life expectancies of 10 years and 1 year, respectively. Female worm fecundity decreases with age [42]. Full model details are provided elsewhere [39, 40] and in Additional File 1: Text S1.

### Simulating immigration

We simulated one-time immigration events of individuals from an area with ongoing transmission to an area free from onchocerciasis. The model was not calibrated to the transmission conditions of a specific real-world focus; rather, we explored a range of parameters to describe a number of archetypal settings. We modelled the transmission intensity to which immigrants would have been subjected prior to relocating to the infection-free community by assuming that in their location of provenance they would have been exposed to an ABR = 10,000 bites/person/year, as an example of a highly endemic setting [24]. This was done with the purpose of generating an appropriate distribution of infection in the ‘source’ population from which to sample worm burden among immigrants, ranging from 0 to 120, with this range motivated by nodulectomy studies conducted in West and Central Africa [43]. We conducted 300 model repeats at this ABR and recorded, for each individual in the source population, their age, sex, equilibrium number of adult *O. volvulus* worms (*W*), microfilarial load (microfilariae/skin snip) and anti-Ov16 serostatus (positive or negative). By simulating immigrants, the dynamics of parasite survival and fecundity of the worms they harbour are correctly transferred upon their ‘arrival’ in the infection-free (‘sink’) population.

We considered infection-free communities, comprising variable population sizes (*N* = 200–1000 individuals) and ABRs (500–1000 bites/person/year), representing communities where either *O. volvulus* had hitherto not been present or had been previously eliminated but where competent black fly vectors occur. We simulated immigration as single events (i.e. immigrants re-locating to a new community), each event consisting of the ‘arrival’ of a given number of *I* immigrants (1–15) harbouring *W* (male and female) worms (0–120) by replacing age- and sex-matched individuals in the infection-free community to maintain a constant population size and structure.

Parameter ranges were selected to span plausible values observed in endemic settings and thus were supported by empirical studies (Table 1, ‘Empirical motivation’ column). These ranges allowed us to explore model sensitivity to parameter values in scenario modelling rather than attempting to capture the conditions in any single focus. Specifically, we motivated our choice of worm burden based on nodulectomy studies which found up to 105 and 130 worms/person in West Africa savannah and forest settings, respectively [43]. Recently, genomic approaches have been used to estimate the number of reproductively active adult worms using sibship analysis, with the number of worms in samples from West and Central Africa ranging from one female and one male per person (asymptotic estimate = 1, 95% confidence interval [CI] 1–1 for each worm sex) to 10 females (asymptotic estimate = 18, 95% CI 10–37) and six males (estimate = 7, 95% CI 6–12) per person [44]. Table 1 describes the parameters used to simulate immigration events, and Additional File 1: Table S1 provides an illustration of the demographic, infection and serology characteristics for *I* = 15 and *W* = 10–20.

**Table 1:** Parameter definitions, values (and motivation) used to simulate single immigration events of individuals from an area with endemic onchocerciasis arriving to an infection-free community

Parameter	Definition	Values	Empirical motivation
*N*	Population size of ‘sink’ infection-free community	200–1000	Typical rural village sizes in OCP [45]
ABR	Annual biting rate of black fly vectors in ‘sink’ infection-free community	500–1000 bites/person/year	Low- to moderate-transmission range [24, 46]
	Annual biting rate of black fly vectors to which immigrants had been exposed in ‘source’ community	10,000 bites/person/year	Selected to generate desired range of adult worm burden [43]
*I*	Number of immigrants	1–15	Migration studies from OCP [32, 33]
*W*	Number of adult *Onchocerca volvulus* (male and female^a^) worms per person harboured by immigrants	0–120	Nodulectomy [43] and genomic [44] studies

^a^EPIONCHO-IBM tracks the number of fertile and non-fertile female worms, as females undergo 3–4 reproductively active cycles per year and alternate between these two states, requiring insemination for each cycle [47]

### Infection persistence and outbreak dynamics

To investigate the probability of infection persistence (i.e. the ‘vulnerability’ of a ‘sink’ community to introduction/re-introduction of infection), we simulated transmission resulting from each single immigration event from the ‘source’ community for 500 years (with this long time horizon being necessary for the system to reach either extinction or endemic equilibrium [48]). For each parameter combination, 300 model runs were conducted. The probability of persistent (endemic) infection was defined as the proportion of runs in which microfilarial prevalence (in all ages) was > 0% at year 500.

To characterise outbreak dynamics, we conducted 300 model runs, each for 100 years, and simulated the mean microfilarial prevalence (in all ages) and the mean anti-Ov16 seroprevalence (in those aged 5–9 years). The outbreak growth rate (intrinsic rate of increase) per annum was calculated using a (non-parametric) numerical approach to compute the derivative (rate of change) of the mean microfilarial prevalence across the 300 model repeats, for each combination of input parameters presented in Table 1. We report both the value of and the number of years to reach the maximum growth rate. Results are also shown for the initial 10 years of simulated outbreaks to illustrate early growth behaviour.

All analyses were conducted using R, version 4.5.1. The EPIONCHO-IBM model code, including user instructions and vignettes, is publicly available at https://github.com/mrc-ide/EPIONCHO.IBM.

We adhered to the five principles of the NTD Modelling Consortium regarding Policy-Relevant Items for Reporting Models in Epidemiology of NTDs (PRIME-NTD), for good practice in NTD modelling [49] (Additional File 1: Text S2, Table S2).

## Results

### Infection persistence

**Fig. 1:**
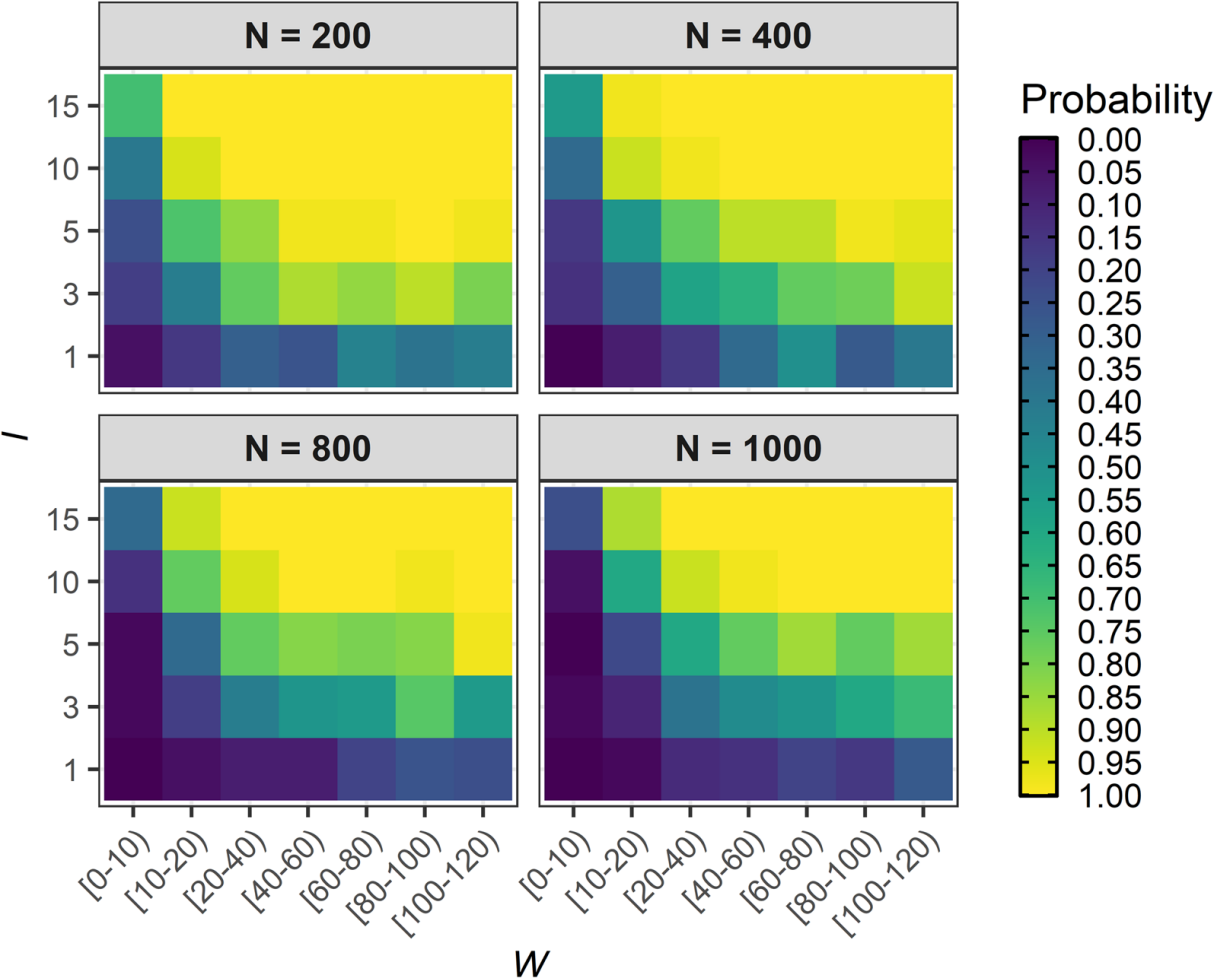
Effect of immigration events on the probability of persistence of *Onchocerca volvulus* transmission in previously infection-free communities. The numbers of immigrants, *I* (*y*-axis), with an adult *Onchocerca volvulus* worm burden, *W* (*x*-axis), are varied, respectively, from 1 to 15 and from 0 to 120, replacing, with immigrant individuals, age- and sex-matched members of an infection-free population experiencing an annual biting rate (ABR) of 500 bites/person/year, exposure heterogeneity, *k*_*E*_ = 0.3, and population size, *N*, of 200, 400, 800 or 1000. The proportion (probability) of persistence for each parameter combination was estimated as the proportion of runs with microfilarial prevalence (all ages) > 0% from 300 repeats of EPIONCHO-IBM simulated for 500 years. The colour scale from dark blue to light yellow indicates an increasing probability of infection persistence

The probability that an immigration event leads to persistent (endemic) *O. volvulus* infection in an infection-free community depends on the ABR (Additional File 1: Fig. S1), the number of immigrants, *I*, their individual worm burden, *W*, and the infection-free community population size, *N*. Figure 1 shows that the proportion of 300 stochastic repeats of EPIONCHO-IBM persisting after 500 years—indicative of endemic transmission—increases with increasing numbers of adult *O. volvulus* worms introduced into the population by immigration (i.e. by increasing *I* and *W*)*.* For example, for *N* = 200, *I* = 1, *W* = 10–20 and ABR = 500, approximately 10–15% of runs persist, whilst when *I* = 15 and *W* = 10–20, 100% of runs persist (Fig. 1). Increasing *N* decreases the probability of persistence for a given value of *I* and *W*. For example, increasing *N* from 200 to 1000, with *I* = 15, *W* = 10–20 and ABR = 500, results in approximately 100% compared to 80–85% persistence, respectively (Fig. 1). This is because of the ‘diluting effect’ of black flies distributing bites over a larger population with a smaller proportion of infected immigrants (7.5% compared to 1.5%)—in agreement with other vector-borne diseases [50]. When the magnitude of incoming immigrants is scaled to the size of the infection-free population (i.e. as a fixed proportion rather than an absolute number), this trend is reversed: increasing *N* leads to *increasing* probability of persistence (due to the weakening effects of stochastic fade-out [48]; Additional File 1: Fig. S2). The impact of increasing the magnitude of inter-individual exposure heterogeneity (reducing *k*_*E*_ from 0.3 to 0.2) for *N* = 400 and ABR = 1000 is shown in Additional File 1: Fig. S3. Increasing the severity of overdispersion in exposure facilitates infection persistence for lower values of *I* and *W*.

### Outbreak dynamics

**Fig. 2:**
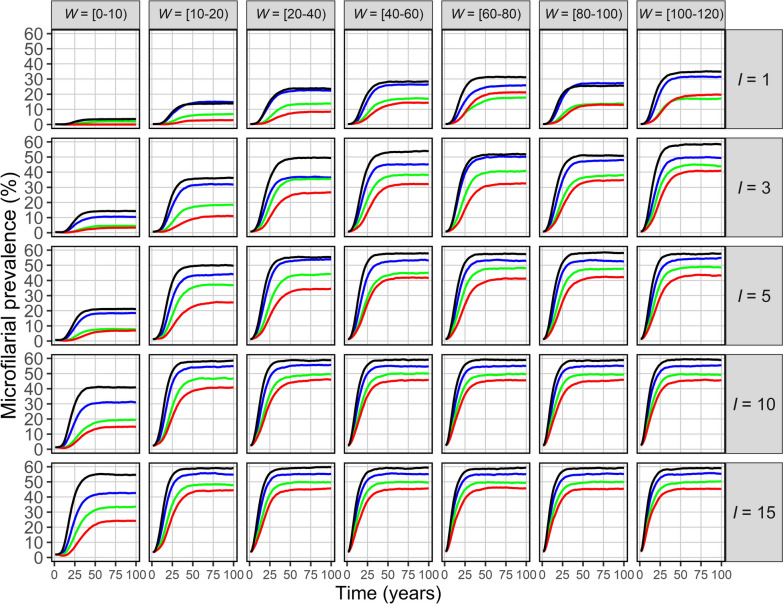
*Onchocerca volvulus* microfilarial prevalence dynamics resulting from infection imported into a previously infection-free community. EPIONCHO-IBM was used to conduct 300 model runs for 100 years varying the numbers of immigrants, *I*, and adult *O. volvulus* worms, *W* (rows and columns, respectively), arriving at an infection-free population of *N* = 400 individuals. Shown is the mean microfilarial prevalence in all ages (*y*-axis) versus time in years (*x*-axis) under four annual biting rate values—500 (red), 600 (green), 800 (blue) and 1000 (black) bites/person/year—with exposure heterogeneity, *k*_*E*_ = 0.3. Microfilarial prevalence indicates the presence of active transmission and the potential for onward transmission (to black fly vectors)

**Fig. 3:**
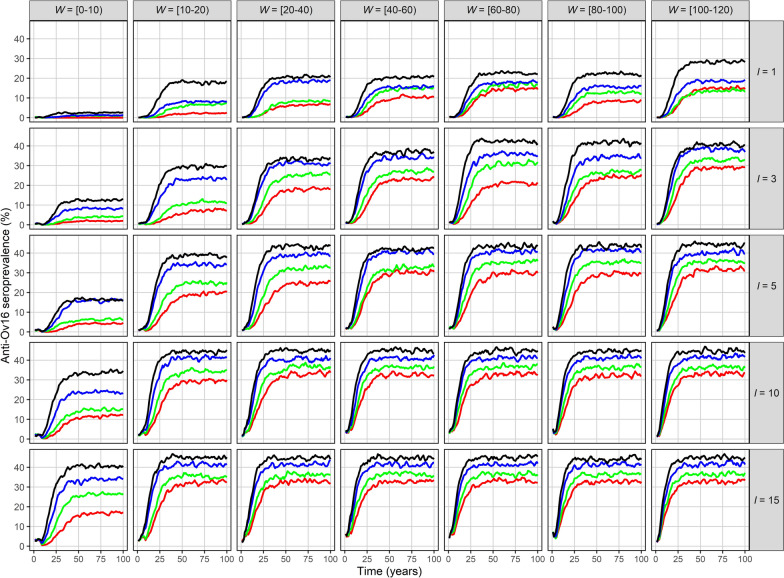
*Onchocerca volvulus* anti-Ov16 seroprevalence dynamics resulting from infection imported into a previously infection-free community. EPIONCHO-IBM was used to conduct 300 model runs for 100 years varying the numbers of immigrants, *I*, and adult *O. volvulus* worms, *W* (rows and columns, respectively), arriving at an infection-free population of *N* = 400 individuals. Shown is the mean anti-Ov16 seroprevalence in those aged 5–9 years (*y*-axis) versus time in years (*x*-axis) under four annual biting rate values—500 (red), 600 (green), 800 (blue) and 1000 (black) bites/person/year—with exposure heterogeneity, *k*_*E*_ = 0.3. Seroprevalence in this age group is an indicator of ongoing transmission [40]

Following introduction of infection by immigration, onchocerciasis outbreak dynamics follow a logistic-type growth pattern towards endemic equilibrium, with the resulting microfilarial prevalence (all ages; Fig. 2) and anti-Ov16 seroprevalence (ages 5–9 years; Fig. 3) depending on ABR, *I* and *W*.

Across all parameter combinations, three main patterns emerge: (i) increasing ABR markedly raises both the equilibrium prevalence and the rate at which endemicity is reached; (ii) higher *W* shortens the lag between importation of parasites and appearance of microfilariae (and of anti-Ov16 antibodies) in the community receiving the immigrants; (iii) larger *I* increases the final magnitude of both microfilarial and anti-Ov16 seroprevalence. Together these factors generate the outcomes presented in Figs. 2 and 3 and the logistic-type trajectories illustrated in Additional File 1: Fig. S4.

In the parameter space considered, low-prevalence persistence typically corresponds to endemic microfilarial prevalence of roughly 5–15% under ABR = 500–600 bites/person/year for moderate immigration events (*I* = 3–5, *W* = 10–20), rising to ~ 20–40% as ABR approaches 800–1000 or as *I* and *W* increase. Persistence is most likely under low ABRs when importation events are higher in magnitude (greater *I* and *W*), with fade-out more likely at lower magnitudes.

For example, for *N* = 400, an immigration event of *I* = 3 and *W* = 10–20 worms generates an endemic microfilarial prevalence (all ages) of approximately 10% and a seroprevalence (in children) of approximately 9% when the ABR = 500. This increases to approximately 45% microfilarial prevalence and 33% seroprevalence if the immigration event is represented by *I* = 15 and *W* = 100–120 (ABR = 500) or up to 60% microfilarial prevalence and 46% seroprevalence when *I* = 15, *W* = 100–120 (ABR = 1000) (Figs. 2, 3).

The maximum growth rate in microfilarial prevalence depends strongly on ABR (and to a lesser extent on *I* and* W*), such that doubling the ABR from 500 to 1000 bites/person/year causes a near-twofold increase (from approximately 0.2 to roughly 0.4 per year) (Additional File 1: Fig. S5). By contrast, the time to maximum growth rate is minimally influenced by ABR. Instead, increasing *W* decreases the time to reach the maximum growth rate across all parameter combinations. Simulations with *W* = 0–10 take over three times as long to reach their maximum microfilarial prevalence growth rate as those with *W* = 100–120 (16 versus 5 years; Additional File 1: Fig. S6).

**Fig. 4:**
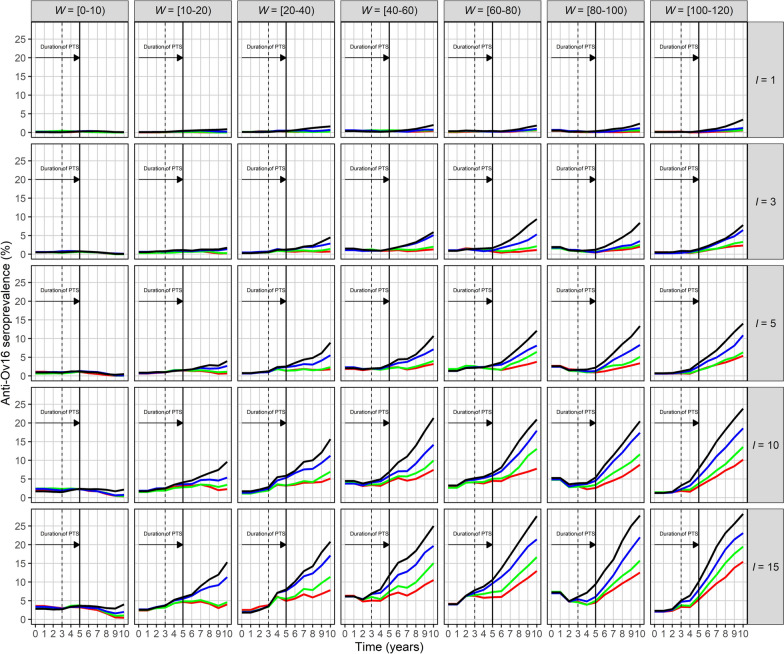
Initial *Onchocerca volvulus* anti-Ov16 seroprevalence dynamics resulting from infection imported into a previously infection-free community. EPIONCHO-IBM was used to conduct 300 model runs for 10 years varying the numbers of immigrants, *I*, and adult *O. volvulus* worms, *W* (denoted by rows and columns, respectively), arriving at an infection-free population (assumed previously onchocerciasis-endemic) of *N* = 400 individuals. The anti-Ov16 seroprevalence in those aged 5–9 years (*y*-axis) is shown for four annual biting rates—500 (red), 600 (green), 800 (blue) and 1000 (black) bites/person/year—with exposure heterogeneity, *k*_*E*_ = 0.3. The post-treatment surveillance (PTS) period is indicated by the vertical dashed line (for 3 years) and the vertical solid line (for 5 years)

After 25 years of simulation, anti-Ov16 seroprevalence in children aged 5–9 years may be as high as 44% when *I* = 15, *W* = 100–120 and ABR = 1000. Immigration events with lower numbers of immigrants, worm burden and ABR values are associated with slower growth rates. When *I* = 3, *W* = 20–40 and ABR = 500, the seroprevalence is about 8% after 25 years (Fig. 3). Under a scenario in which the community was previously onchocerciasis-endemic and had reached EOT, for a PTS period of 3–5 years, the seroprevalence in 5–9-year-olds would be < 5% at 3 years and < 10% at 5 years of PTS in nearly all simulated scenarios, and < 5% in all simulations with up to *I* = 5 immigrants, irrespective of worm burden or ABR (Fig. 4).

## Discussion

We have investigated the vulnerability of ‘sink’ communities free from onchocerciasis to introduction/re-introduction of *O. volvulus* from immigrants arriving from a ‘source’ area with ongoing transmission (with an ABR = 10,000 bites/person/year) using the individual-based transmission model, EPIONCHO-IBM. We show that onchocerciasis introduction/re-introduction and persistence can take place when biting rates are low (500 bites/person/year, less than two bites per day), particularly when communities are small and receive an influx of infected immigrants of about 4–8% even when immigrants harbour relatively low worm burden (Fig. 1). We also show that the risk of onchocerciasis outbreaks in infection-free communities can be high even with modest black fly vector densities, which is exacerbated by larger importation events into small communities (Figs. 2, 3).

For a community to be vulnerable to infection introduction/re-introduction, it must be ‘ecologically receptive’, have a suitable population size and demographic structure, and receive sufficient parasite importation through immigration. Ecological receptivity requires proximity to productive black fly breeding sites and the presence of competent vector species at biting densities high enough to support transmission. These bites must be distributed (according to exposure patterns) across a community of a particular size and structure, which influence the likelihood that introduced parasites will become established and persist. In addition, the community must receive enough infected immigrants from endemic areas to introduce a sufficient worm burden. However, increased exposure heterogeneity facilitates persistence at lower numbers of immigrants and smaller worm burden. These factors interact to determine whether importation leads to fade-out or persistent endemic transmission. In what follows, we discuss each of these components in turn, placing our modelling results in the context of empirical observations.

Operationally, vulnerable settings are those where (i) competent vectors and productive breeding sites occur near settlements with ABR of ≈ 500 to 1000 bites/person/year, (ii) communities are small (≈ 200 to 400 people) such that the proportion of immigrants relative to the population of the recipient community is sizeable, and (iii) there is documented influx or seasonal migration from endemic foci (even if infection load of immigrants is low). These criteria correspond closely to the low-prevalence persistence conditions identified by our model.

The risk of re-introduction of transmission in communities with higher ABRs will clearly be greater and will remain so for many years after elimination has been achieved [51]. The vulnerability of receptive communities will also depend on the local vector(s): different *Simulium* species vary in their vector competence and anthropophagy, with some members of *S. damnosum* s.l. being particularly efficient vectors [52, 53]. The presence of suitable breeding sites close to human settlements is also critical [30, 54].

Demographic characteristics further shape vulnerability. Smaller communities are particularly at risk because black fly bites are distributed among fewer individuals. This contrasts with predictions for directly transmitted pathogens [55]. For example, our model predicts that communities of 200 individuals—typical of rural communities across SSA, with a mean of 285 individuals per village in the OCP database [45]—would have greater than 90% probability of yielding persistent endemic transmission from just 15 infected immigrants harbouring moderate (10–20) worm burden. Heterogeneity in individual exposure further exacerbates this effect.

Human movement determines the likelihood of parasite importation into receptive communities. An example is provided by human migration studies conducted in the OCP, where river valleys had been protected by 14–15 years of vector control, and biting rates were low. As antivectorial operations were about to stop and black flies were allowed to return, there was concern that human migration from non-controlled onchocerciasis-endemic foci would lead to a relapse of transmission [32]. In Burkina Faso, the immigrant population (from Côte d’Ivoire) ranged from 0 to 18% of the population in the villages, with an average of 5%. The prevalence of infection in immigrants was 8%, and 2% of these had microfilarial loads greater than 16 microfilariae/skin snip. The study concluded that human migration had caused importation of *O. volvulus* from non-controlled areas [32]. A more recent study in villages across three river basins, also in Burkina Faso, found that 30 immigrant workers had returned from a highly endemic region in Côte d'Ivoire with mean microfilarial loads ranging from 0.5 to 73 (8.1 on average) microfilariae/skin snip. One individual with no travel history was also recorded as infected, indicating that autochthonous transmission could already have been rekindled [33]. In the example presented in Additional File 1: Table S1, the simulated microfilarial load of immigrants ranged from 0 to 19 microfilariae per skin snip.

We further illustrate that small importation events could generate protracted, slowly increasing microfilarial prevalence and anti-Ov16 seroprevalence dynamics which may be difficult to detect, while larger importation events may yield more rapidly increasing and overt outbreaks (Figs. 3 and 4). These findings have important practical implications for programmatic decision-making on the duration and type of surveillance activities that will be required in foci under PTS and PES, or elsewhere where black fly vectors have the opportunity to establish productive breeding sites and biting populations.

The long lifespan of *O. volvulus* (10 years on average [42]) results in protracted outbreak dynamics following importation of infection. For example, in the scenario where 10 immigrants harbouring 10–20 worms arrive in a community with an ABR of 500, it takes about 25 years for the anti-Ov16 seroprevalence in 5–9-year-olds to reach 15% and another 25 years to double. This can present a challenge to the timely detection of onchocerciasis outbreaks, with infection going unnoticed until severe clinical manifestations become evident. For instance, in northern Uganda, internally displaced populations relocated to onchocerciasis-endemic areas in close proximity to black fly breeding sites during the Lord’s Resistance Army civil war from 1986 to 2006, which compromised security and the ability to implement onchocerciasis control. The number of incident cases of nodding syndrome (in the OAE spectrum) rose from 0 in 1989 to 50 in 2000 and more than 300 by 2011, prompting a nodding syndrome outbreak investigation in 2012 [30]. In South Sudan, the construction of the Maridi Dam took place in the mid-1950s, providing a suitable breeding ground for black fly vectors, but it was not until the 1990s that children began to present with OAE, and by 2019 the anti-Ov16 seroprevalence in under-10-year-olds had reached 24% overall (95% exact CI 18–32%) and 41% (95% CI 29–54%) in the communities closest to the dam [54]. Therefore, passive surveillance approaches based on clinical manifestations alone will not be sufficient to prevent outbreaks from leading to established infection.

Current WHO guidelines [37] propose 3–5 years of PTS. However, according to our results, this would only be appropriate for detecting emerging outbreaks deriving from large importation events in communities with higher ABRs. Our model predicts that in many circumstances, anti-Ov16 seroprevalence in children may still be low 5 years after an immigration event. Hence, PTS and PES will likely require longer durations of active surveillance to be confident that re-introduction of infection has not occurred, notwithstanding the outstanding questions on whether current serological diagnostics are adequate for detecting low-seroprevalence signals of emerging infection [56].

Complementary activities to serological (and entomological) surveillance should also be considered. For example, signals of positive serology should be accompanied by investigations on migration patterns, particularly when positive cases have arrived from foci where transmission is known to be ongoing [32, 33]. Local patterns of migration among small rural communities within endemic ‘transmission zones’ should also be further investigated so that PTS and PES activities can be coordinated at larger geographical scales (which could constitute ‘epidemiological units’) over which migration and movement take place. This should also apply to decisions on stopping interventions and verification of EOT, as it may be unwise for interventions to be halted in a particular focus until EOT in the wider transmission zone has been demonstrated. The delineation of transmission zones remains challenging, but progress has been made using genomic approaches to quantify the extent and direction of black fly movement and migration patterns among (within- and between-country) endemic foci [57, 58]. These challenges will also apply to cross-border transmission zones where inter-governmental collaboration will be essential for coordinated decision-making on stopping interventions, initiating surveillance and potentially detecting positive cases (in humans and/or flies) that may warrant further investigation and prompt response [59]. Inadequate cooperation will render many border areas extremely vulnerable to importation events [60].

In accordance with observational epidemiological studies [61, 62], our results suggest that low ABRs can sustain transmission at low prevalence. This does not preclude the possibility that persistent low prevalence could be sustained via continuous importation from higher-transmission areas [63] (we considered only single importation events). Modelling studies using ONCHOSIM have suggested that low-level transmission can persist when residents of communities with low endemicity travel (for visiting or working reasons) to foci with higher endemicity and return home [25].

Under the conditions explored, infection may remain stable over long timescales despite relatively modest vector biting rates, with fade-out occurring mainly when importation is rare or the imported infection load is low. This highlights the fact that sustained transmission can be ignited by importation of infection under epidemiological conditions that might appear inconsequential, with these results complementing previous modelling of transmission thresholds and hypoendemic stability in the absence of infection importation at biting rates lower than those explored here [48]. Our results therefore suggest that communities may remain epidemiologically receptive to renewed transmission in the presence of immigration. Even modest ABRs in receptive areas thus warrant continued surveillance, consistent with WHO guidelines [37] and previous recommendations that any detected transmission signal should prompt investigation and response [48].

## Conclusions

We have explored, using EPIONCHO-IBM, the vulnerability of communities free from *O. volvulus* to importation of infection. We show that low numbers of immigrants with modest worm burden may be sufficient to seed an onchocerciasis outbreak with potential for persistent transmission. We also show that the protracted outbreak dynamics that result from the long adult *O. volvulus* life expectancy, incubation and pre-patent periods will mean that prolonged periods of active surveillance will be required to ensure sustained, country-wide EOT. As we aim towards a post-elimination era for NTDs, outbreak dynamics—seldom considered for such long-enduring endemic diseases—must be better understood to inform the design of robust surveillance activities. This is of utmost importance as integration of NTD control, elimination and surveillance into the broader context of country health systems will become imperative under the current global health financing situation [64], and as we progress towards 2030 and beyond.

## Supplementary Information

Additional file1 (PDF 855 KB)

## Data Availability

Data supporting the main conclusions of this study are included in the manuscript.

## Abbreviations

ABR: Annual biting rate
APOC: African Programme for Onchocerciasis Control
CI: Confidence interval
DALY: Disability-adjusted life year
EOT: Elimination of transmission
ESPEN: Expanded Special Project for Elimination of Neglected Tropical Diseases
MDA: Mass drug administration
NTD: Neglected tropical disease
OAE: Onchocerciasis-associated epilepsy
OCP: Onchocerciasis Control Programme in West Africa
PES: Post-elimination surveillance
PTS: Post-treatment surveillance
s.l.: Sensu lato
UI: Uncertainty interval
WHO: World Health Organization

## Code availability

The EPIONCHO-IBM model code is available at https://github.com/mrc-ide/EPIONCHO.IBM.
