# Supplementary material for: Modelling the effects of immigration on the re-introduction of onchocerciasis
Source: Parasit Vectors. 2026 Jan 14;19:53. doi: 10.1186/s13071-025-07213-z (PMC12849067; doi:10.1186/s13071-025-07213-z)
Supplement: Supplementary file 1 — Additional file1 (PDF 855 KB) [file 13071_2025_7213_MOESM1_ESM.pdf]

Supplementary Information

Additional File 1. Supplementary methods and results

Modelling the effects of immigration on the re-introduction of onchocerciasis

Jacob N. Stapley[1*], Maria-Gloria Basáñez[1*], Aditya Ramani[1,2], Martin Walker[1,2,†] and Jonathan I.D. Hamley[3,4,†]

[1] MRC Centre for Global Infectious Disease Analysis and London Centre for Neglected Tropical Disease Research, Department of Infectious Disease Epidemiology, School of Public Health, Imperial College London, London, UK

[2] Department of Pathobiology and Population Sciences, Royal Veterinary College, Hatfield, UK

[3] Multidisciplinary Center for Infectious Diseases, University of Bern, Switzerland

[4] Department of Visceral Surgery and Medicine, Inselspital, Bern University Hospital, University of Bern, Switzerland

*Correspondence: Jacob N. Stapley (j.stapley20@imperial.ac.uk) and Maria-Gloria Basáñez (m.basanez@imperial.ac.uk)

† Joint Senior Authors

Text S1. Incorporation of anti-Ov16 seroprevalence as an output of EPIONCHO-IBM

Ramani et al. have incorporated anti-Ov16 seroprevalence as an output of EPIONCHO-IBM [1]. Each individual in the modelled population has an anti-Ov16 serostatus (positive or negative) according to their infection status as described in the following. Briefly, Ramani et al. tested several hypotheses regarding which *Onchocerca volvulus* parasite stage(s) elicit(s) seroconversion (defined as the production of immunoglobulin G4 (IgG4) antibody responses against the Ov16 *O. volvulus* antigen) with the aim of determining the timing of seroconversion following infection. The hypotheses tested were that seroconversion takes place: H1) following exposure to infective, L3 larvae; H2) following the moult from L4 larvae to L5, juvenile adults; H3) following the establishment of a mature worm (of either sex), and H4) following the production of microfilariae by a mating worm pair. The hypothesis that seroconversion is triggered by patent infection (H4) provided a better quantitative fit to data from 4,257 individuals living in 58 ivermectin-naïve communities in Gabon [2]. Hypothesis H4 is also consistent with the results of a study investigating anti-Ov16 dynamics following experimental infection of non-human primates with *O. volvulus* L3 larvae [3]. Therefore, for the work presented here, we used hypothesis H4 (that IgG4 antibody seroconversion in response to Ov16 is elicited by a mating worm pair producing microfilariae). For this *in silico* study, we further assumed that the Ov16 test has perfect sensitivity and specificity.

Table S1. Example of modelled demographic, infection and serology characteristics for *I* = 15 immigrants and *W* = 10-20 worms from a 'pool' of immigrants exposed to 10,000 bites/person/year, and exposure heterogeneity, k_E = 0.3, simulated with 300 model repeats of EPIONCHO-IBM after reaching equilibrium.

Immigrant (*I*)	Age (yr)	Sex	Microfilariae per skin snip	Adult worms (*W*)	Anti-Ov16 serostatus
1	9	Female	8.00	14	1
2	39	Female	1.00	12	1
3	11	Male	4.00	20	1
4	69	Male	14.00	20	1
5	17	Male	0.25	14	1
6	5	Female	0.25	17	1
7	20	Male	1.00	16	1
8	5	Female	3.75	15	1
9	29	Male	1.25	19	1
10	72	Female	4.25	13	1
11	10	Female	0.00	11	0
12	33	Female	3.50	20	1
13	18	Male	19.00	20	1
14	19	Male	3.50	18	1
15	60	Female	1.00	16	1

Text S2. Modelling for policy: PRIME-NTD

For the analyses presented, we adhered to the Five Principles of the Neglected Tropical Diseases (NTD) Modelling Consortium for good practice in policy-relevant NTD modelling [4]. Table S2 briefly describes the five tenets, how they were fulfilled, and where in the Main Text and/or Additional File 1 they can be found.

Table S2. Policy-Relevant Items for Reporting Models in Epidemiology of Neglected Tropical Diseases (PRIME-NTD) summary table [4].

**Principle**	**What has been done to satisfy the principle?**	**Where in the manuscript is this described?**
**Stakeholder engagement**	Discussions with modelling and policy-focused collaborators	Author list, Acknowledgements section
**Complete model documentation**	References to the full description of EPIONCHO-IBM are provided. An Open Access link to the code has been given	Methods section and Code availability statement
**Complete description of data used**	This was an *in silico* study which did not use raw data for the modelling presented. The range of worm burdens used for immigrants was motivated by nodulectomy and genomic studies	Data availability statement. Main Text, Table 1 of Main Text and References
**Communicating uncertainty**	Sensitivity analyses were conducted by varying the number of immigrants (I), their worm burdens (W), the size of the infection-free community (N), and its annual biting rate (ABR), with an example given for varying exposure heterogeneity (k_E)	Table 1 in Methods. Figures 1 to 4 and their captions in Results. Additional File Figures S1 to S6
**Testable model outcomes**	Results of immigration studies are discussed. The dynamics of anti-Ov16 seroprevalence in children could be compared with data from post-treatment surveillance studies	Results and Discussion

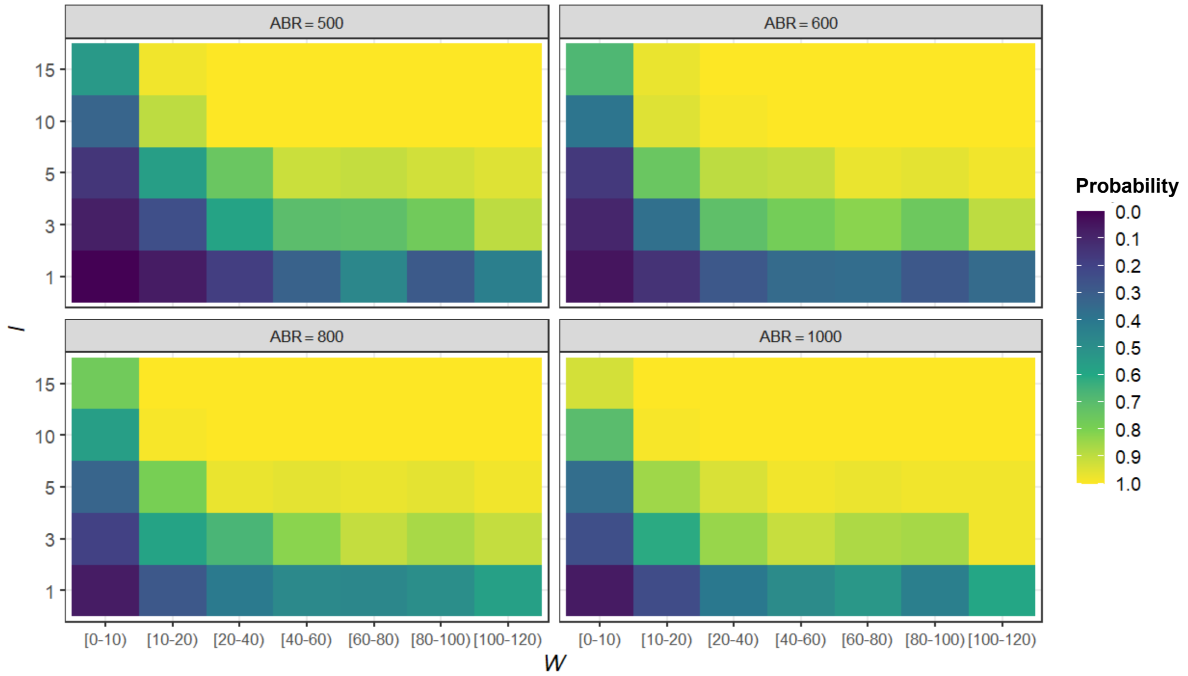

Figure S1. Effect of annual biting rate (ABR) on the probability of persistence of *Onchocerca volvulus* transmission in previously infection-free communities. The numbers of immigrants, *I* (*y*-axis) with an adult *Onchocerca volvulus* worm burden, *W* (*x*-axis) are varied, respectively, from 1 to 15 and from 0 to 120, replacing, with immigrant individuals, age- and sex-matched members of an infection-free population experiencing an annual biting rate (ABR) of 500, 600, 800 and 1000 bites/person/year, with a population size, *N*, of 400 individuals, and exposure heterogeneity, k_E = 0.3. The proportion (probability) of persistence for each parameter combination was estimated as the proportion of runs with microfilarial prevalence (all ages) >0% from 300 repeats of EPIONCHO-IBM simulated for 500 years. The colour scale from dark blue to light yellow indicates an increasing probability of infection persistence.

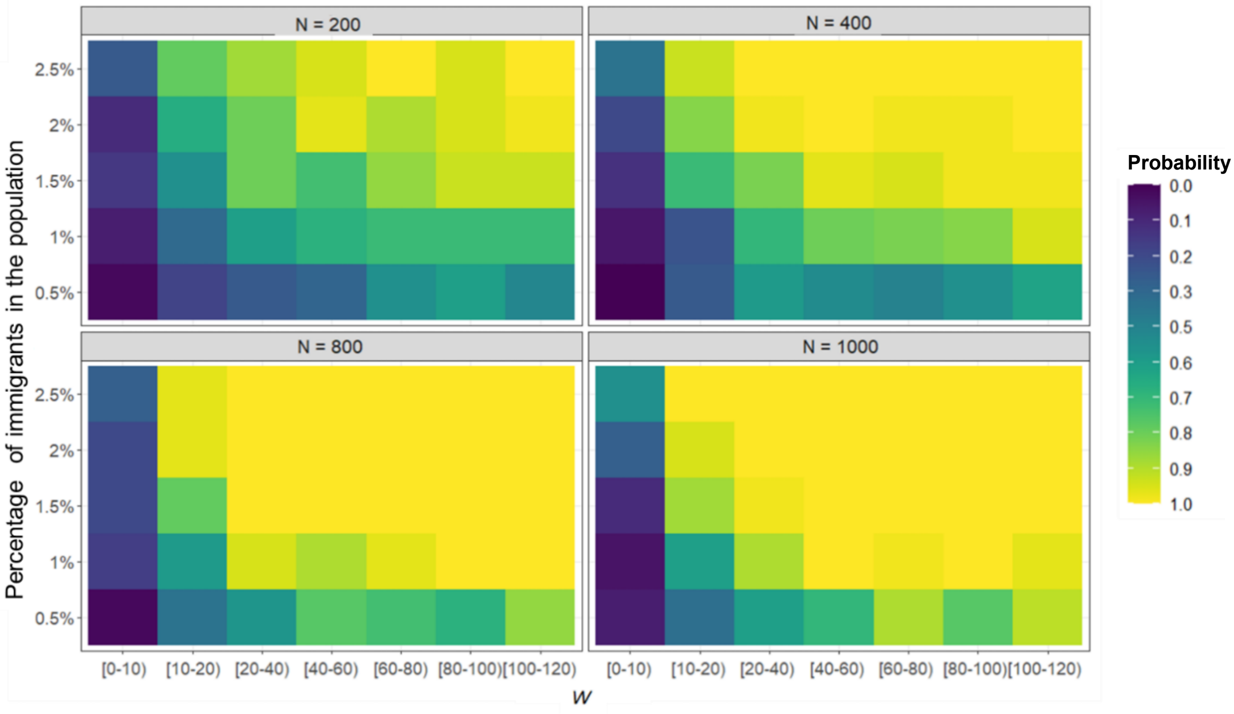

Figure S2. Effect of varying the proportion of immigrants on the probability of persistence of *Onchocerca volvulus* transmission in previously infection-free communities. The proportion (in percent) of immigrants (*y*-axis) with an adult *Onchocerca volvulus* worm burden, *W* (*x*-axis) are varied, respectively, from 0.5% to 2.5% and from 0 to 120, replacing, with immigrant individuals, age- and sex-matched members of an infection-free population experiencing an annual biting rate (ABR) of 500 bites/person/year, with a population size, *N*, of 200, 400, 800 and 1000 individuals, and exposure heterogeneity, k_E = 0.3. For *N* = 200 the number of immigrants, *I*, varies from 1 to 5; for *N* = 400 from 2 to 10; for *N* = 800 from 4 to 20, and for *N* = 1000 from 5 to 25. The proportion (probability) of persistence for each parameter combination was estimated as the proportion of runs with microfilarial prevalence (all ages) >0% from 300 repeats of EPIONCHO-IBM simulated for 500 years. The colour scale from dark blue to light yellow indicates an increasing probability of infection persistence.

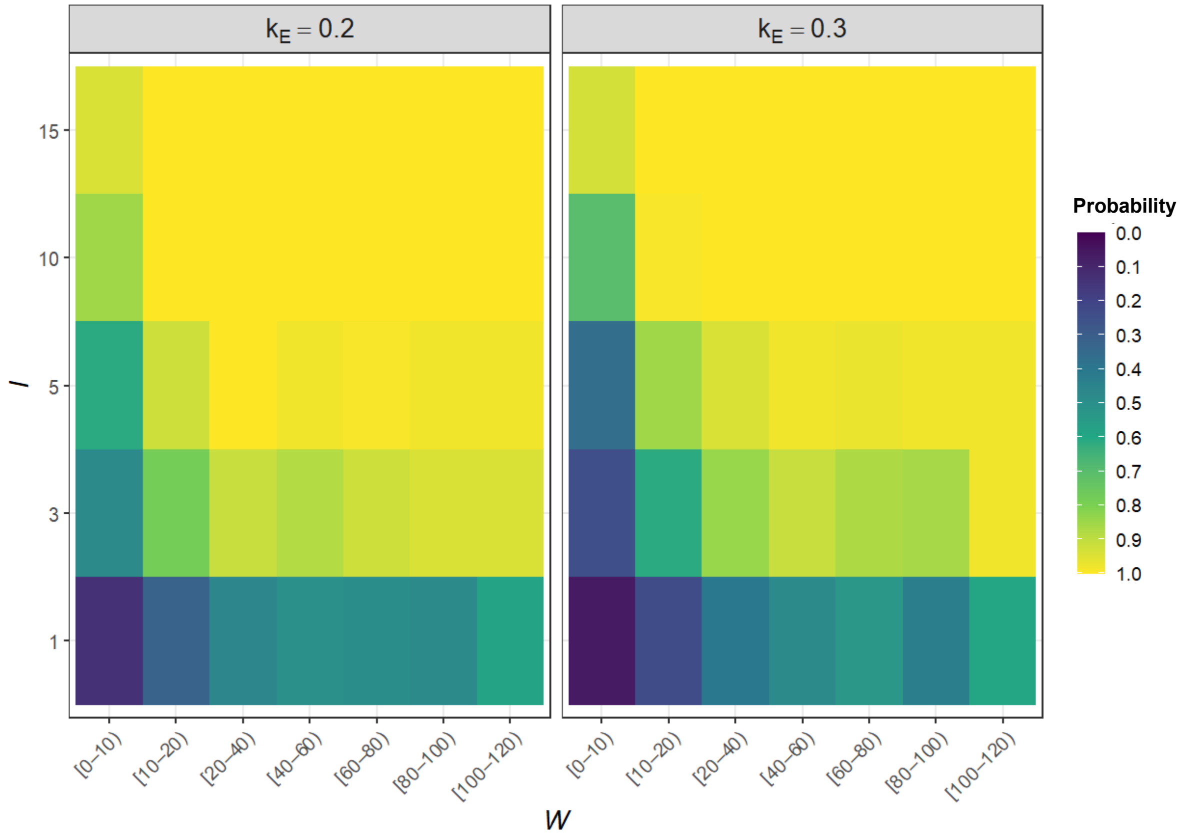

Figure S3. Effect of individual-level exposure heterogeneity (k_E) on the probability of persistence of *Onchocerca volvulus* transmission in previously infection-free communities. The numbers of immigrants, *I* (*y*-axis) with an adult *Onchocerca volvulus* worm burden, *W* (*x*-axis) are varied, respectively, from 1 to 15 and from 0 to 120, replacing, with immigrant individuals, age- and sex-matched members of an infection-free population experiencing a k_E = 0.2 (left panel) or k_E = 0.3 (right panel), with a population size, *N*, of 400 individuals and an annual biting rate (ABR) of 1000 bites/person/year. A decreasing value of k_E corresponds to increased exposure heterogeneity. The proportion (probability) of persistence for each parameter combination was estimated as the proportion of runs with microfilarial prevalence (all ages) >0% from 300 repeats of EPIONCHO-IBM simulated for 500 years. The colour scale from dark blue to light yellow indicates an increasing probability of infection persistence.

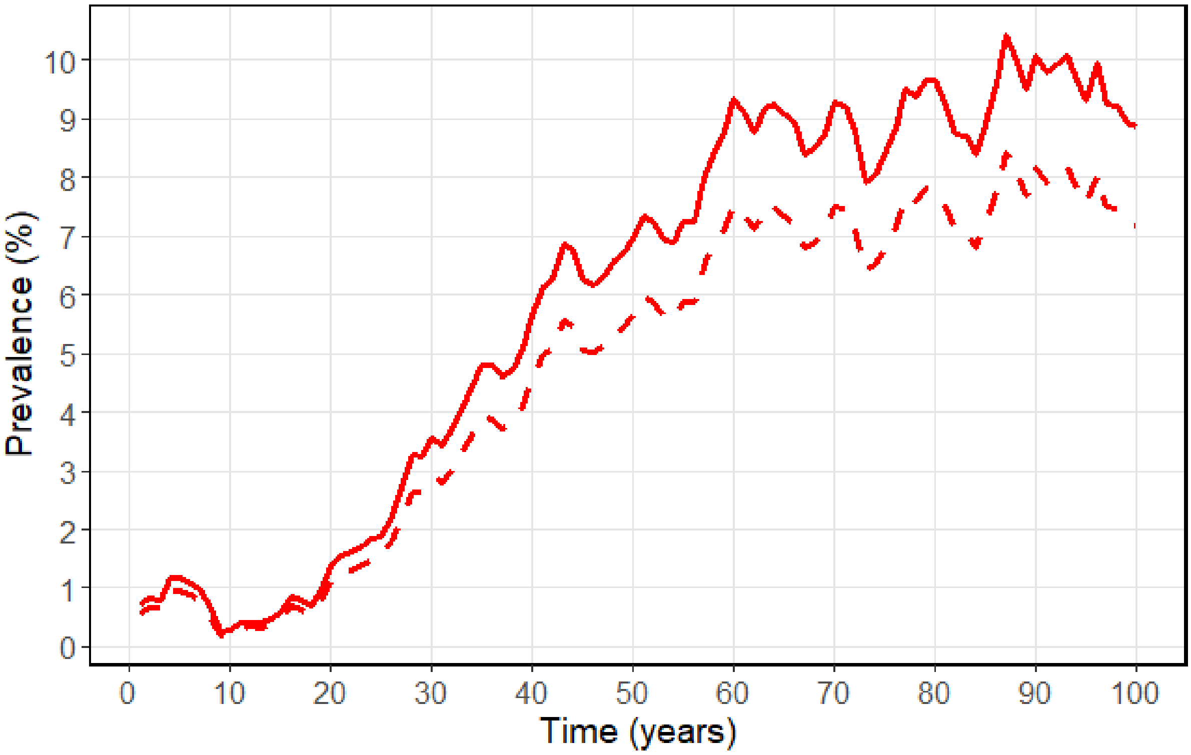

Figure S4. Example *Onchocerca volvulus* dynamics resulting from infection imported into a previously infection-free community. Three hundred runs of EPIONCHO-IBM with three immigrants, *I*, each with 10-20 adult *O. volvulus* worms, *W,* replacing age- and sex-matched members of a previously infection-free population (*N* = 400), experiencing an annual biting rate (ABR) of 500 bites/person/year, and exposure heterogeneity, k_E = 0.3, were run for 100 years. Mean microfilarial prevalence (all ages, red solid line) and mean anti-Ov16 seroprevalence (children aged 5-9 years, red dashed line) are shown for this example parameter combination. See Fig. S5 and Fig. S6 for the full range of parameter combinations explored in this analysis.

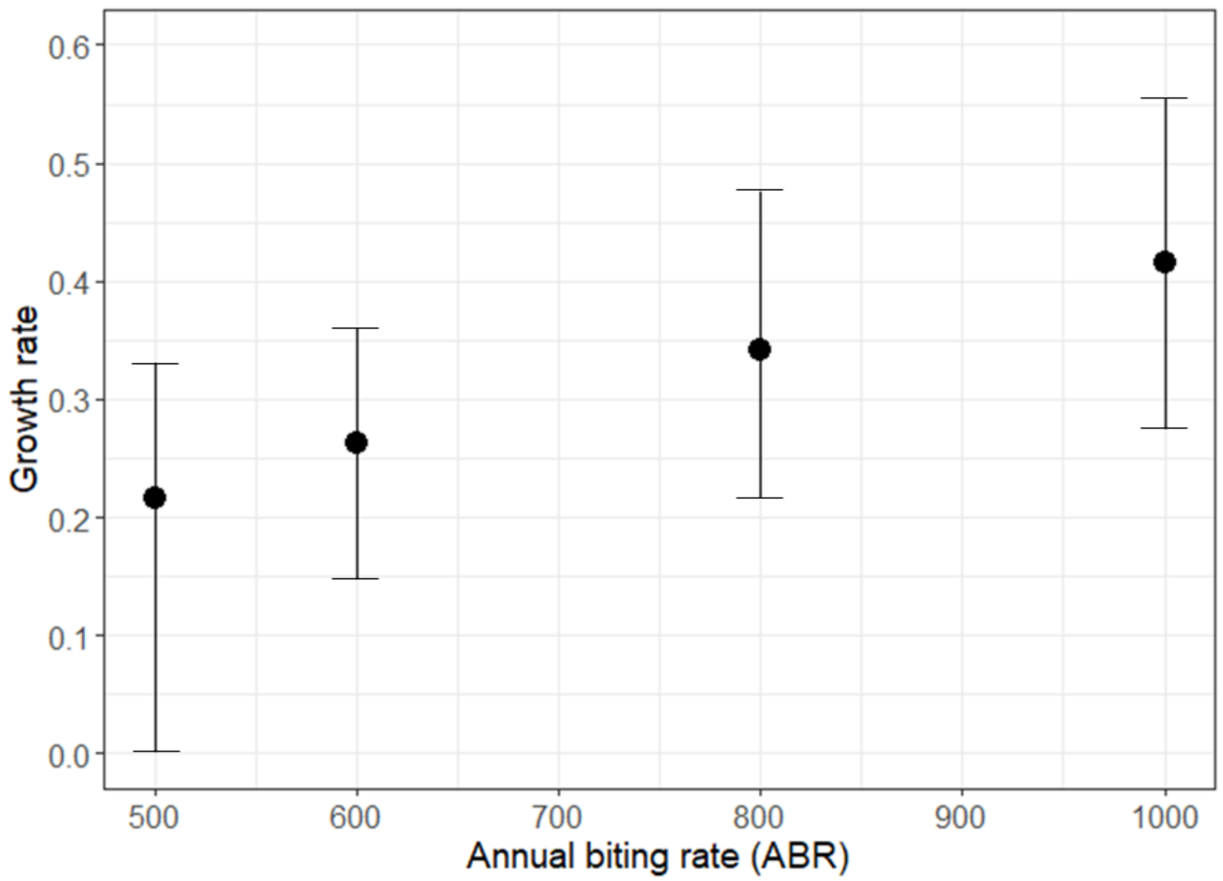

Figure S5. Effect of annual biting rate (ABR) on the maximum intrinsic rate of increase (growth rate, per annum) of *Onchocerca volvulus* microfilarial prevalence outbreaks following immigration events into infection-free communities. The maximum annual rate of increase in microfilarial prevalence (*y*-axis) was calculated for each modelled population (*N* = 400) following the (re-)introduction of infection via immigration of individuals from an area with ongoing transmission (ABR = 10,000 bites/person/year). For each ABR (500 to 1000) in the infection-free community (*x*-axis), 300 model repeats were performed across a range of immigration scenarios, from *I* = 1 to 15 and *W* = 0 to 120, with exposure heterogeneity, k_E = 0.3 (Table 1 of Main Text). Black circles indicate the mean of the maximal annual microfilarial prevalence growth rates, averaged across immigration scenarios and simulation repeats; vertical bars indicate the range of model outputs.

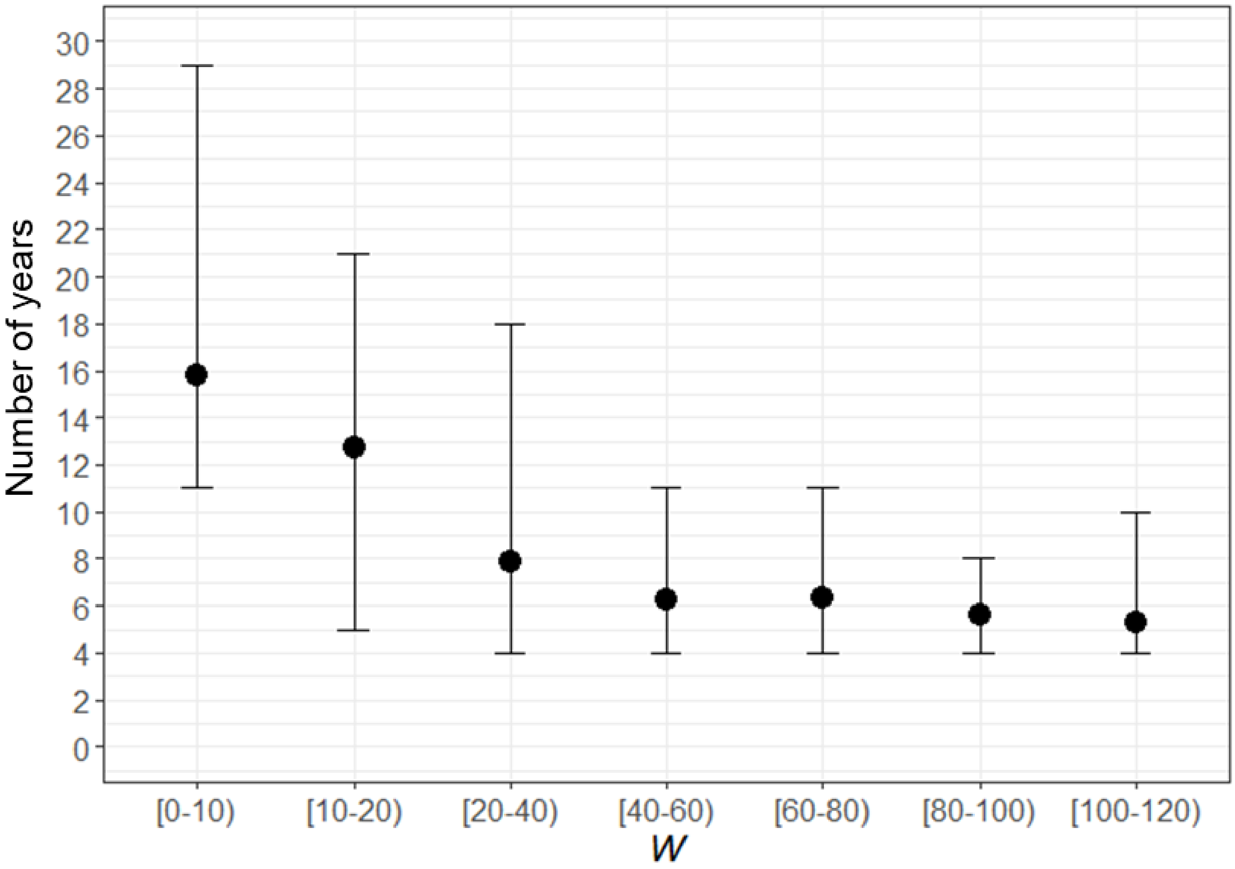

Figure S6. Effect of immigrant worm burden, *W*, on the number of years required to attain the maximum intrinsic rate of increase (growth rate, per annum) of *Onchocerca volvulus* microfilarial prevalence outbreaks following immigration events into infection-free communities. The number of years to reach the maximum growth rate (*y*-axis) was calculated for each modelled population (*N* = 400) following the (re-)introduction of infection via immigration of individuals from an area with ongoing transmission (ABR = 10,000 bites/person/year). For each worm burden (0-10 to 100-120) of immigrants arriving in the infection-free community (*x*-axis), 300 model repeats were performed across a range of immigration scenarios, with *I* = 1 to 15, ABR = 500 to 1000, and exposure heterogeneity, k_E = 0.3 in the infection-free community. Black circles indicate the mean number of years required to attain the maximum yearly growth rate of microfilarial prevalence, averaged across immigration scenarios and simulation repeats; vertical bars indicate the range of model outputs.

Supplementary References

1. Ramani A, Stapley JN, Dixon MA, Hamley JID, Amaral L-J, Basáñez M-G, et al. Modelling onchocerciasis seroprevalence for control and elimination. Nature Comms. (under review). Pre-print available at Research Square. 2025. https://www.researchsquare.com/article/rs-7140160/v1. Accessed 10 November 2025.
2. Atsame J, Stapley JN, Ramani A, Mourou R, Ntsame E, Efame E, Angue ON, Obiang JL, Pilotte N, Gass K, Basáñez MG. Comparison of diagnostic tools to assess the feasibility of programmatic use of rapid diagnostic tests for onchocerciasis: a dataset from Gabon. Data Brief. 2024;57:110901.
3. Cama VA, McDonald C, Arcury-Quandt A, Eberhard M, Jenks MH, Smith J, Feleke SM, Abanyie F, Thomson L, Wiegand RE, Cantey PT. Evaluation of an Ov-16 IgG4 enzyme-linked immunosorbent assay in humans and its application to determine the dynamics of antibody responses in a non-human primate model of *Onchocerca volvulus* infection. Am J Trop Med Hyg. 2018;99:1041–48.
4. Behrend MR, Basáñez MG, Hamley JID, Porco TC, Stolk WA, Walker M, de Vlas SJ, NTD Modelling Consortium. Modelling for policy: the five principles of the Neglected Tropical Diseases Modelling Consortium. PLoS Negl Trop Dis. 2020;14:e0008033.
